# Magnesium Sulfate Treatment During Total Knee Arthroplasty Decreases Postoperative Urinary Retention: A Retrospective Propensity Score-Matched Analysis

**DOI:** 10.3390/jcm9030620

**Published:** 2020-02-25

**Authors:** Jin-Woo Park, Eun-Kyoung Kim, Dongsik Lim, Tak Kyu Oh, Seongjoo Park, Sang-Hwan Do

**Affiliations:** 1 Department of Anesthesiology and Pain Medicine, Seoul National University Bundang Hospital, Seongnam-si 13620, Korea; jinul8282@gmail.com (J.-W.P.); dsklim3@naver.com (D.L.); airohtak@hotmail.com (T.K.O.); struka@snubh.org (S.P.); 2 Department of Pain Clinic, Bundang Jesaeng Hospital, Seongnam 13590, Korea; eunkyoung2lovely@gmail.com; 3 Department of Anesthesiology and Pain Medicine, College of Medicine, Seoul National University, Seoul 03080, Korea

**Keywords:** magnesium sulfate, postoperative urinary retention, spinal anesthesia, total knee arthroplasty

## Abstract

Postoperative urinary retention (POUR) is a common complication after total knee arthroplasty. Currently, there are no clinical data regarding the effects of magnesium sulfate on POUR. Here, we investigated the effects of intraoperative magnesium sulfate infusion in patients with POUR following total knee arthroplasty. We reviewed the medical records of patients who underwent elective unilateral total knee arthroplasty under spinal anesthesia between June 2016 and May 2018. The patients were grouped based on whether they were treated with magnesium (magnesium group) or not (control group). We investigated the incidence of POUR and the postoperative analgesic requirement. Totally, 483 patients were included in the analysis. After propensity score matching with the control group, the magnesium group showed a lower incidence of POUR (odds ratio, 0.49; 95% CI, 0.29–0.83; *p* = 0.011) and lesser opioid consumption (*p* = 0.049) than the control group. Multivariate logistic regression analysis revealed that intraoperative continuous infusion of magnesium (*p* = 0.008) and age (*p* = 0.001) were significantly related to the incidence of POUR. This retrospective observational study demonstrated that administration of magnesium sulfate was associated with a lower incidence of POUR following total knee arthroplasty.

## 1. Introduction

Postoperative urinary retention (POUR) is a common complication after anesthesia and surgery. Especially, the incidence of POUR has been reported to be relatively high following lower limb joint replacement surgeries [1,2,3]. Although spinal anesthesia is reported to be superior to general anesthesia for these procedures [4,5], urinary retention following replacement surgery occurs more frequently with spinal anesthesia than with general anesthesia [1]. POUR results in pain or discomfort in the lower abdomen as well as secondary side effects such as urinary tract infections. Furthermore, it is mainly treated with urethral catheterization, which can be highly inconvenient for the patients and induce complications such as catheter-related infections and urethral injuries [1,3,6].

Magnesium sulfate is an N-methyl-D-aspartate (NMDA) antagonist and an effective adjuvant for postoperative pain [7,8,9]. Through previous studies, we have demonstrated that intraoperative infusion of magnesium sulfate during total knee arthroplasty (TKA) decreases postoperative pain and opioid analgesic requirements [7,10]. Systemic opioid usage is a well-known risk factor for POUR [1,11,12,13]. Additionally, magnesium also attenuates the sympathetic tone [9,14]. The sympathetic nervous system affects bladder relaxation and internal sphincter contraction [1,11]. Therefore, treatment with magnesium may have a facilitating effect on postoperative urination. However, there are currently no clinical data regarding the effects of magnesium sulfate on POUR.

Therefore, in this study, we aimed to compare the incidence of POUR after TKA with spinal anesthesia between patients who were treated with magnesium sulfate during surgery and those who were not. We hypothesized that the incidence of POUR would be lower in patients treated with magnesium sulfate.

## 2. Materials and Methods

This retrospective observational study was approved by the Institutional Review Board (IRB) of Seoul National University Bundang Hospital (IRB number, B-1809-493-111; approval date, 11 September 2018). The requirement for informed consent was waived by the IRB due to the retrospective nature of the analysis.

### 2.1. Study Population

We reviewed the electronic medical records of patients aged 19 years or older who underwent elective unilateral TKA under spinal anesthesia at the Seoul National University Bundang Hospital between June 2016 and May 2018. Patients who received general or epidural anesthesia, those with preoperatively inserted Foley catheters, chronic kidney disease with a glomerular filtration rate less than 45 mL min^−1^ (stage ≥3b), an American Society of Anesthesiologists (ASA) physical status ≥3, preoperative voiding difficulty, and incomplete or missing medical records were excluded from this study. If a patient underwent staged bilateral TKA during the study period, data from the first TKA were solely utilized.

### 2.2. Spinal Anesthesia and Magnesium Administration

Patients were routinely premedicated with intravenous midazolam at the preoperative holding area. For spinal anesthesia, an optimal dose of 0.5% hyperbaric bupivacaine with 10–20 μg fentanyl was injected intrathecally. The target level of the sensory block was T10. After determining the proper sensory block level and achieving hemodynamic stability, patients were asked if they wanted to be sedated during the surgery, and intravenous sedation was provided according to the patients’ request. Propofol or dexmedetomidine was utilized to sedate the patients at the discretion of the anesthesiologist as follows: (1) propofol sedation, target-controlled infusion of propofol was maintained within an effect-site concentration of 0.5–2.0 μg·mL^−1^; (2) dexmedetomidine sedation, 1 μg·kg^−1^ dexmedetomidine was loaded over 10 min and then administered continuously (0.1–0.5 μg·kg^−1^·h^−1^). The effect-site concentration or continuous infusion rate was controlled to achieve a Modified Observer’s Assessment of Alertness/Sedation scale 3 or 4. The anesthesiologist also decided whether to administer magnesium sulfate during surgery. The protocol for the intraoperative magnesium infusion was as follows: (1) magnesium group, magnesium sulfate was loaded (50 mg·kg^−1^) over 15 min during anesthesia induction and then continuously infused (15 mg·kg^−1^·h^−1^) until the end of the operation; and (2) control group, no magnesium infusion. In the patients with severe renal insufficiency, bradycardia, atrioventricular block (≥2nd degree), and heart failure, magnesium administration was contraindicated [15,16]. A Foley catheter is not routinely inserted for patients undergoing unilateral TKA at Seoul National University Bundang Hospital.

### 2.3. Postoperative Pain Management

For postoperative pain management, intravenous patient-controlled analgesia (PCA) was initiated after the surgery and maintained during postoperative days 0–3. The PCA regimen, based on the patient’s weight and comorbidities, was as follows: 8–15 μg·mL^−1^ fentanyl (according to the age of the patient); total volume, 100 mL; bolus dose, 1 mL without basal continuous infusion; and lockout interval, 10 min. Standard postoperative medications (650 mg oral acetaminophen, 200 mg celecoxib, and 75 mg pregabalin) were routinely prescribed every 12 h after surgery, and rescue analgesics (50 mg intravenous ketoprofen, 100 mg intravenous tramadol, or 50 mg intravenous meperidine) were administered per the patient’s request. Before initiating the spinal anesthesia, an ipsilateral femoral nerve catheter was placed under ultrasound guidance, and a postoperative continuous femoral nerve block was administered with 0.2% ropivacaine infused through a catheter at a rate of 4 mL·h^−1^ until 48 h after the operation.

### 2.4. Study Outcomes

In our hospital, POUR was defined as the inability to void within 6 hours or a post-void residual urine (bladder scan volume) >300 mL, therefore requiring urinary catheterization. Incidence of POUR during the first 3 postoperative days was the primary measured outcome of this retrospective study. Demographic data (age, sex, and body mass index), preoperative comorbidities (ASA class, hypertension, diabetes mellitus, ischemic heart disease, cerebrovascular disease, anemia, and glomerular filtration rate), anesthesia data (intrathecal bupivacaine and fentanyl, intravenous sedation, and intraoperative magnesium), and operational characteristics (operation time, estimated blood loss, and intraoperative fluid) were also collected. After 72 postoperatively, intravenous PCA was disconnected and the usage of fentanyl was reported. The amounts of opioids administered for PCA and rescue analgesia in postoperative days 0–3 were aggregated and calculated as a morphine equivalent dose.

### 2.5. Statistical Analysis

Continuous variables are expressed as the mean with standard deviations. Categorical variables are shown as numbers (%). Student’s *t*-test was utilized to compare continuous variables, and chi-square or Fisher’s exact test was used to compare categorical variables as appropriate. We performed a univariate logistic regression to analyze the association between each variable and POUR. Variables with *p* < 0.2 from the univariate analysis were included in the following multivariate logistic regression analysis.

Propensity score matching was performed to minimize the risk of selection bias and confounder effects between the two groups. The patients were matched at a 1:1 ratio by the nearest-neighbor method. Propensity scores were calculated with a logistic regression analysis, and included covariates were demographics, comorbidities, anesthetic characteristics, and operation data. To determine the balance between the two groups, we tested the standardized mean difference for each covariate. After propensity score matching, McNemar’s test or paired t-test were used for analyses, as appropriate. SPSS version 21.0 software (SPSS Inc., IBM, Chicago, IL, USA) was utilized for all statistical analyses; a two-sided *p*-value of less than 0.05 was considered statistically significant.

## 3. Results

**Figure 1:**
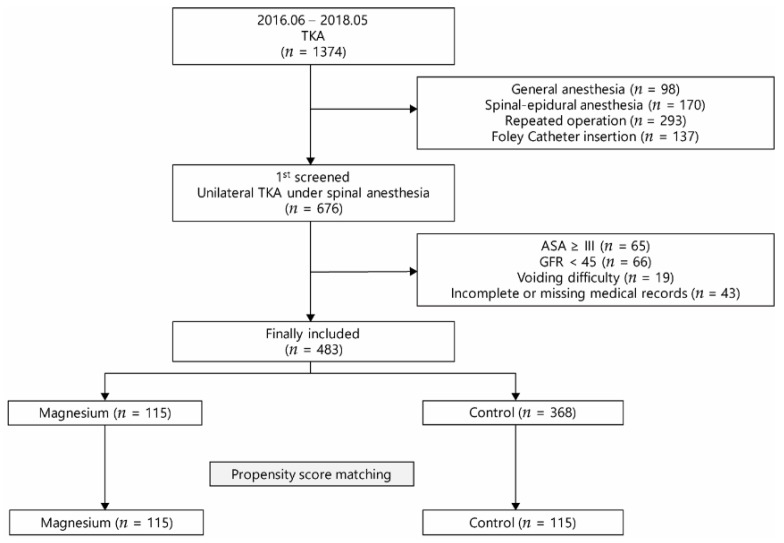
Flow chart of patient selection. TKA, total knee arthroplasty; ASA, American Society of Anesthesiologists; GFR, glomerular filtration rate.

A total of 1374 patients underwent TKA from June 2016 to May 2018. Among them, 483 patients were finally included in the analysis. Magnesium sulfate was intraoperatively administered in 115 patients, and 368 patients did not receive magnesium treatment during the surgery. After propensity score matching, 115 patients were assigned to each group (Figure 1). A comparison of the baseline characteristics before and after the propensity score matching is shown in Table 1. Standardized mean differences of all covariates were less than 0.1 after matching, indicating a good balance.

Of the original 483 patients, the incidence of POUR was significantly lower in the magnesium group than in the control group (odds ratio, 0.60; 95% CI, 0.39–0.91; *p* = 0.015; Table 2). Postoperative opioid analgesia was also significantly less in the magnesium group compared to the control group (estimated difference, 34.75; 95% CI, 5.42–64.07; *p* = 0.020; Table 2). Similar results were obtained after propensity score matching. The magnesium group showed a lower incidence of POUR (odds ratio, 0.49; 95% CI, 0.29–0.83; *p* = 0.011) and lesser opioid consumption (estimated difference, 36.76; 95% CI, 1.72–72.02; *p* = 0.049) than the control group (Table 2).

According to univariate regression analysis, intraoperative magnesium infusion (odds ratio, 0.60; 95% CI, 0.39–0.91; *p* = 0.016) and age of patients (odds ratio, 1.04; 95% CI, 1.02–1.07; *p* = 0.002) were significantly related to the incidence of POUR (Table 3). A multivariate logistic regression analysis also revealed that magnesium continuous infusion (odds ratio, 0.56; 95% CI, 0.37–0.86; *p* = 0.008) and age (odds ratio, 1.05; 95% CI, 1.02–1.07; *p* = 0.001) were also significant factors associated with POUR (Table 4). The multivariate model showed proper goodness of fit as assessed with the Hosmer–Lemeshow test (*p* = 0.173).

## 4. Discussion

This retrospective study demonstrated that intraoperative magnesium sulfate treatment is associated with a lower incidence of POUR following TKA. The analysis was strengthened by the propensity score matching, and our results suggested that continuous infusion of magnesium sulfate during TKA reduces the risk of POUR as well as the requirements for postoperative analgesic management. To the best of our knowledge, this is the first clinical study to confirm that magnesium sulfate treatment can facilitate postoperative urination.

The incidence of POUR following lower limb joint arthroplasty ranges widely from 11% to 84% [1,3,12]. This wide range may be related to different patient populations, surgical procedures, and anesthetic conditions. According to previous studies, POUR is more frequent in men than in women and increases with the patient’s age [1,17,18]. Spinal anesthesia, longer duration of surgery, and excessive infusion of intravenous fluid are also known risk factors of POUR [1,17,19,20,21]. Like previous studies, we confirmed that age was significantly associated with POUR. However, the regression analysis did not show any correlation between the incidence of POUR and other known risk factors, which might have been because of the small sample size of this study or the fact that we collected and analyzed the medical data of patients undergoing unilateral TKA with spinal anesthesia only in a single tertiary medical center. Anesthetic and surgical conditions were generally similar among all the patients, making it difficult to reveal statistically significant associations between these variables and the incidence of POUR. Moreover, in the Korean elderly population, women are at much greater risk of requiring TKA compared to men [22]. A small sample size of male patients might make it difficult to confirm any gender differences in POUR.

Magnesium confers anti-nociceptive effects by inhibiting the NMDA receptors. Intraoperative intravenous administration of magnesium sulfate has been reported to reduce postoperative pain and/or opioid consumption [7,9,23], as we confirmed in the current study. Systemic opioids inhibit acetylcholine release from the parasympathetic sacral nerves, which control detrusor tone and thereby increase the risk of POUR [24]. We hypothesized that the reduction in postoperative opioid analgesic requirement due to magnesium sulfate administration may be effective in decreasing the incidence of POUR. However, TKA is a highly painful surgery, and most patients in the magnesium group as well as in the control group required a lot of analgesic medications after surgery. Although the continuous infusion of magnesium sulfate was associated with somewhat decreased opioid consumption, we failed to reveal statistically significant associations between opioid consumption and the incidence of POUR. Meanwhile, magnesium infusion was found to be significantly associated with POUR by a multivariate analysis. Therefore, magnesium sulfate seemed to alleviate the risk for POUR after TKA through different functions, and not simply by reducing opioid consumption after surgery.

Magnesium attenuates sympathetic tone by inhibiting N-type calcium channels at peripheral sympathetic nerve endings and by directly blocking the catecholamine receptors [9,14,25]. As the sympathetic system causes bladder relaxation and internal sphincter contraction, magnesium treatment might allow for bladder contraction and internal sphincter relaxation, thereby facilitating postoperative urination [1,11]. Contrary to our hypothesis, some preclinical studies have reported that magnesium could suppress detrusor muscle contractility by inhibiting calcium channels in the smooth muscle [26,27], suggesting that magnesium might interfere with urination. However, these preclinical results were obvious with high concentrations of magnesium ions (5 mM) or with accompanying low concentrations of calcium ions (0.6 mM). Magnesium is normally obtained only through diet, and following a major surgery, the serum magnesium levels readily decrease [28,29]. The incidence of hypomagnesemia was reported to be 19% before cardiac operations, to peak to 71% after surgery, and to subside to 66% at 24 h postoperatively [30]. Through previous clinical trials, our magnesium treatment regimen resulted in increased postoperative magnesium concentrations up to 1.3–1.6 mM without any detectable complications of hypermagnesemia, and the increased concentration returned to normal level within postoperative day 1–3 [23,28]. Fortunately, this level of magnesium concentration helps with postoperative urination, according to our results. The increased magnesium concentration returned to normal level.

Micturition is an interaction among complex neural pathways [31,32]. As many surgical or anesthetic factors can interfere with the physiological balance and result in lower urinary tract dysfunction [1,11], the mechanism of POUR has not been completely understood. Therefore, it is very important to be aware of and to identify the risk factors of POUR and to make efforts to prevent it. With a steady increase of outpatient surgeries, treatment with urethral catheterization would rather be restrictive [1]. Therefore, every attempt to decrease the incidence of POUR in various clinical situations should be continued.

There are a few limitations to be considered in this study. First, given the retrospective nature of this study, the magnesium treatment protocol was not randomized and thus, there might be a selection bias. To minimize this bias, we utilized propensity score matching to balance the characteristics between the two study groups. Second, the etiology of POUR is multifactorial and complex; thus, there might have been some unknown confounding factors that affected our results despite the propensity score matching adjustment. Third, we investigated postoperative opioid consumption as an indicator of pain management, although ketorolac was also utilized for rescue analgesia. However, patients receiving ketorolac accounted for a relatively small portion of patients receiving postoperative analgesia, and all analgesics except ketorolac could be summed up in morphine equivalent doses and compared between the groups. Finally, we analyzed the data at a single medical center; therefore, the generalizability of this study may be limited.

In conclusion, this retrospective observational study demonstrated that magnesium sulfate treatment was associated with a lower incidence of urinary retention following TKA. The postoperative pain-relieving effects of magnesium have also been confirmed in numerous previous studies. This study may provide additional clue for magnesium treatments in improving postoperative recovery. Our findings should be confirmed in further prospective analysis.

## Figures and Tables

**Table 1:** Baseline characteristics before and after propensity score matching.

	Unmatched Cohort (*n* = 483)				Matched Cohort (*n* = 330)			
	Control *n* = 368	Magnesium *n* = 115	SMD	*p*	Control *n* = 115	Magnesium *n* = 115	SMD	*p*
Age, year	71.2 (7.2)	72.2 (7.3)	0.137	0.217	72.2 (7.4)	72.2 (7.3)	<0.001	0.921
Sex								
Male	51 (13.9)	16 (13.9)	0.002	0.988	15 (13.0)	16 (13.9)	0.026	>0.999
Female	317 (86.1)	99 (86.1)			100 (87.0)	99 (86.1)		
BMI, kg·m^−2^	27.0 (3.6)	26.5 (3.3)	0.142	0.126	26.8 (3.7)	26.5 (3.3)	0.075	0.141
ASA status (I/II)								
I	48 (13.0)	7 (6.1)	0.220	0.040	7 (6.1)	7 (6.1)	<0.001	>0.999
II	320 (87.)	108 (93.9)			108 (93.9)	108 (93.9)		
Hypertension	240 (65.2)	82 (71.3)	0.129	0.227	87 (75.7)	82 (71.3)	0.099	0.551
Diabetes mellitus	105 (28.5)	25 (21.7)	0.154	0.152	26 (22.6)	25 (21.7)	0.021	>0.999
IHD *	10 (2.7)	2 (1.7)	0.063	0.740	3 (2.6)	2 (1.7)	0.060	>0.999
CVD *	16 (4.3)	8 (7.0)	0.120	0.261	6 (5.2)	8 (7.0)	0.073	0.791
Anemia (Hb <10 g·dL^−1^)	6 (1.6)	1 (0.9)	0.064	>0.999	1 (0.9)	1 (0.9)	<0.001	>0.999
GFR, mL·min^−1^	81.8 (14.8)	83.6 (13.1)	0.125	0.206	82.7 (14.7)	83.6 (13.1)	0.065	0.621
Spinal anesthesia								
Bupivacaine, mg	11.7 (1.3)	12.0 (1.3)	0.231	0.069	11.9 (1.2)	12.0 (1.3)	0.024	0.850
Intrathecal FTN, μg	15.7 (7.2)	13.4 (5.7)	0.335	0.002	13.6 (5.0)	13.4 (5.7)	0.040	0.764
Operative characteristics								
Operation time, min	130.3 (28.0)	128.6 (31.4)	0.059	0.587	128.1(26.8)	128.6 (31.4)	0.016	0.899
Estimated blood loss, mL	72.1 (66.2)	80.4 (65.9)	0.126	0.243	80.2 (79.8)	80.4 (65.9)	0.002	0.986
Intravenous fluid, mL	448.5 (164.8)	446.1 (165.7)	0.015	0.892	442.6 (152.1)	446.1 (165.7)	0.022	0.871
Premedication								
Midazolam, mg	2.3 (1.0)	2.0 (1.0)	0.300	0.004	2.1 (1.0)	2.0 (1.0)	0.098	0.141
Sedation								
None	204 (55.4)	62 (53.9)	0.423	<0.001	66 (55.7)	62 (53.9)	0.098	0.382
Propofol	117 (31.8)	2 (1.7)			5 (3.5)	2 (1.7)		
Dexmedetomidine	47 (12.8)	51 (44.3)			44 (40.9)	51 (44.3)		

BMI, body mass index; ASA, American Society of Anesthesiologists; IHD, ischemic heart disease; CVD; cerebrovascular disease; GFR, glomerular filtration rate; FTN, fentanyl. * Accidentally found mild vascular stenosis without affecting daily functioning (New York Heart Association functional class 1).

**Table 2:** Postoperative urinary retention and postoperative analgesia in POD 0 to 3, before and after propensity score matching.

	Control	Magnesium	Estimated Difference (95% CI)	Odds Ratio (95% CI)	*p*
Before matching					
Urinary retention	217/368 (59.0)	53/115 (46.1)		0.60 (0.39–0.91)	0.015
MEC	240.3 (140.1)	205.5 (136.7)	34.8 (5.4–64.1)		0.020
After matching					
Urinary retention	73/115 (63.5)	53/115 (46.1)		0.49 (0.29–0.83)	0.011
MEC	242.4 (133.8)	205.5 (136.7)	36.9 (1.7–72.0)		0.049

POD, postoperative day; MEC, morphine equivalent consumption.

**Table 3:** Results of univariate analysis of variables associated with urinary retention.

	Odds Ratio (95% CI)	*p*
Magnesium continuous infusion	0.60 (0.39–0.91)	0.016
Age	1.04 (1.02–1.07)	0.002
Sex		
Male	1	
Female	0.67 (0.39–1.15)	0.143
BMI	0.98 (0.93–1.04)	0.559
ASA		
I	1	
II	0.98 (0.56–1.7)	0.941
DM	1.26 (0.84–1.90)	0.271
IHD *	1.11 (0.35–3.54)	0.864
CVD *	1.61 (0.68–3.85)	0.280
Anemia	0.31 (0.06–1.62)	0.165
Bupivacaine	1.02 (0.89–1.17)	0.752
Intrathecal FTN	1.01 (0.98–1.04)	0.482
Operation time	1.00 (0.99–1.00)	0.475
Estimated blood loss	1.00 (1.00–1.00)	0.788
Intravenous fluid	1.00 (1.00–1.00)	0.375
Premedication (midazolam, mg)	1.01 (0.78–1.30)	0.947
Sedation		
None	1	
Propofol	0.97 (0.63–1.50)	0.887
Dexmedetomidine	0.67 (0.41–1.08)	0.096
Morphine equivalent consumption in POD 0 to 3	1.00 (1.00–1.00)	0.368

BMI, body mass index; ASA, American Society of Anesthesiologists; DM, diabetes mellitus; IHD, ischemic heart disease; CVD, cerebrovascular disease; FTN, fentanyl; POD; postoperative day. * Accidentally found mild vascular stenosis without affecting daily functioning (New York Heart Association functional class 1).

**Table 4:** Results of multivariate analysis of variables associated with urinary retention.

	Odds Ratio (95% CI)	*p*
Magnesium continuous infusion	0.56 (0.37–0.86)	0.008
Age	1.05 (1.02–1.07)	0.001
Sex		
Male	1	
Female	0.63 (0.37–1.10)	0.102
Anemia	0.27 (0.05–1.48)	0.132
Sedation		
None	1	
Propofol	0.96 (0.61–1.52)	0.869
Dexmedetomidine	0.81 (0.48–1.38)	0.439
